# Facile synthesis of Fe_3_O_4_@pyrogallol-formaldehyde resin@Ag core–shell nanomaterials for the catalytic degradation of contaminants

**DOI:** 10.1039/d5ra02755a

**Published:** 2025-07-08

**Authors:** Liping Jiang, Yang Xi, Ziyi Xu, Zewen Song, Yuwei Cui, Haijun Zhou

**Affiliations:** a School of Materials Science and Engineering, Jiangsu University of Science and Technology Zhenjiang China zhouhaijun@just.edu.cn

## Abstract

Noble metal nanoparticles (NPs) show excellent performance in catalysis, but their strong aggregation effect can lead to a decrease in or even disappearance of their catalytic activity. In this study, Fe_3_O_4_@pyrogallol-formaldehyde resin@Ag (Fe_3_O_4_@PGFR@Ag) nanomaterials were synthesized using Fe_3_O_4_ as a magnetic core and pyrogallol-formaldehyde resin (PGFR) as a shell layer. The presence of Fe_3_O_4_ ensured rapid material recovery. At the same time, the phenolic hydroxyl group in PGFR enabled the *in situ* reduction of Ag^+^ to form embedded Ag NPs, effectively avoiding the aggregation and shedding of Ag NPs. Cetyltrimethylammonium bromide (CTAB) was used to modify the surface charge of the catalyst. Results showed that negatively charged Fe_3_O_4_@PGFR@Ag exhibited high catalytic activity, with a 90% higher catalytic rate constant for cationic dye rhodamine B (RhB) compared with Fe_3_O_4_@PGFR@Ag-CTAB. Positively charged Fe_3_O_4_@PGFR@Ag-CTAB showed high catalytic activity, with a 124% higher catalytic rate constant for the anionic dye methyl orange (MO) compared with Fe_3_O_4_@PGFR@Ag. Therefore, the matching of the charges of the catalyst and contaminants, which facilitates the adsorption of the pollutants around the catalyst, has a significant impact on the catalytic performance and should be considered in the process of pollutant treatment.

## Abstract 2

In this study, Fe_3_O_4_@PGFR@Ag was synthesized using Fe_3_O_4_ as a magnetic core and pyrogallol-formaldehyde resin (PGFR) as a shell layer. Fe_3_O_4_@PGFR@Ag exhibited high catalytic activity for RhB. Fe_3_O_4_@PGFR@Ag-CTAB showed high catalytic activity for MO.

## Graphical abstract

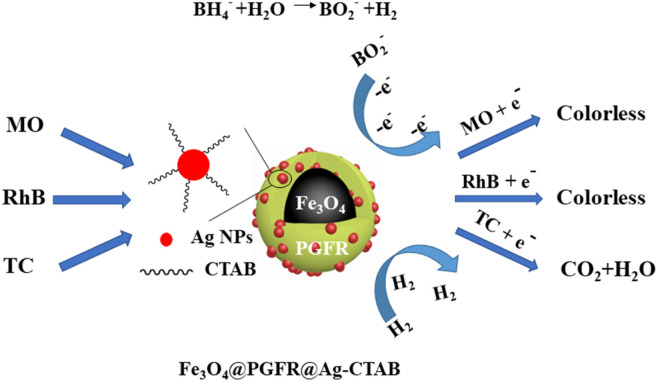

## 1. Introduction

The rapid development of modern industries has brought great convenience to humanity, but it has resulted in serious water pollution.^1–3^ Many organic dyes exhibiting persistent biotoxicity are difficult to degrade and enter the natural water circulation system *via* industrial wastewater, posing a significant threat to human survival and the sustainable and healthy development of the natural environment.^4–7^ Anionic dyes such as methyl orange (MO), which is widely used in leather and wool production; rhodamine B (RhB), used in the cosmetics industry; and tetracycline (TC), used in the medical sector, can pose a serious threat to the health of the natural environment and the survival of flora and fauna if improperly treated.^8,9^ Therefore, organic dye removal and rapid decolorization have become important research topics. Noble metal nanoparticles have been identified as an effective means to achieve this goal and have attracted much attention in the past few decades.^10–13^

According to reports, noble metal nanoparticles have good catalytic selectivity for organic pollutants and have attracted extensive attention from researchers in the past few decades.^14–16^ Ag nanoparticles (NPs) can quickly realize electron transfer in a catalytic system, accelerating the catalytic hydrogenation process on the surface of organic pollutants, ultimately leading to the decolorization of organic pollutants such as RhB and MO.^17–20^ However, the strong aggregation tendency of Ag nanoparticles makes them easily agglomerate, resulting in a decrease in or even disappearance of their catalytic activity.^21–28^ Moreover, individual nanoparticles are difficult to separate from the catalytic system, making it impossible to achieve controlled recycling of the catalytic material. Therefore, magnetic core–shell materials that can be quickly recycled and stably loaded onto noble metal nanoparticles have attracted researchers' attention.^29–35^

In this study, we designed Fe_3_O_4_@PGFR@Ag nanomaterials, which are composed of magnetic Fe_3_O_4_ as a core material and pyrogallol-formaldehyde resin (PGFR) as a shell material. The presence of magnetic core materials ensures the rapid recovery of nanoparticles after catalysis. PGFR possesses strong adhesion and easy surface modification properties. During the reduction of AgNO_3_, the phenolic hydroxyl groups on the surface of PGFR enable the *in situ* reduction of AgNO_3_ without external reducing agents. By contrast, in silicon-based material systems, the reduction of AgNO_3_ requires the addition of external reducing agents, such as hydrazine hydrate. Fe_3_O_4_@PGFR@Ag exhibits excellent catalytic activity for the cationic dye RhB. Therefore, nanomaterials whose surface charges are switched using CTAB exhibit efficient catalytic activity toward anionic dyes.

## 2. Experimental section

### 2.1 Materials and chemicals

Iron(iii) chloride hexahydrate (FeCl_3_·6H_2_O), trisodium citrate dihydrate, ethylene glycol, anhydrous sodium acetate, pyrogallol, a formaldehyde solution (37–40%), an ammonium solution (25–28%), silver nitrate, sodium borohydride (NaBH_4_), cetyltrimethylammonium bromide, rhodamine B (RhB), tetracycline (TC), and methyl orange (MO) were purchased from Sinopharm Chemical Reagent Co., Ltd. All chemicals were used as received.

### 2.2 Synthesis of Fe_3_O_4_

The synthesis of Fe_3_O_4_ nanoparticles was performed using the following procedure.^36^ Briefly, 1.08 g of FeCl_3_·6H_2_O and 0.46 g of trisodium citrate were added to 40 mL of ethylene glycol to form a dispersed solution by stirring at room temperature. Then, 2.4 g of anhydrous sodium acetate was added to the above solution. The above solution was stirred for 30 min and then transferred to a closed polytetrafluoroethylene reactor for 12 h at 200 °C. Then, the reactor was cooled to room temperature. The black precipitate was collected using a magnetic block and repeatedly washed with deionized water and ethanol. Then, the obtained black powder was dried under vacuum at 60 °C for 6 h.

### 2.3 Synthesis of Fe_3_O_4_@PGFR

The prepared Fe_3_O_4_ particles (0.08 g) and pyrogallol (0.1637 g) were uniformly dispersed in 200 mL of a deionized water solution containing 142 μL of ammonia and stirred well. Then, a formaldehyde solution (222 μL) was added to the solution, dispersed evenly by stirring for 30 minutes, heated to 80 °C, and maintained for 30 minutes. After the completion of the reaction, Fe_3_O_4_@PGFR was thoroughly washed with deionized water and ethanol, separated using a magnet, and dried under vacuum for 6 h.

### 2.4 Synthesis of Fe_3_O_4_@PGFR@Ag and Fe_3_O_4_@PGFR@Ag-CTAB

**Fig. 1:**
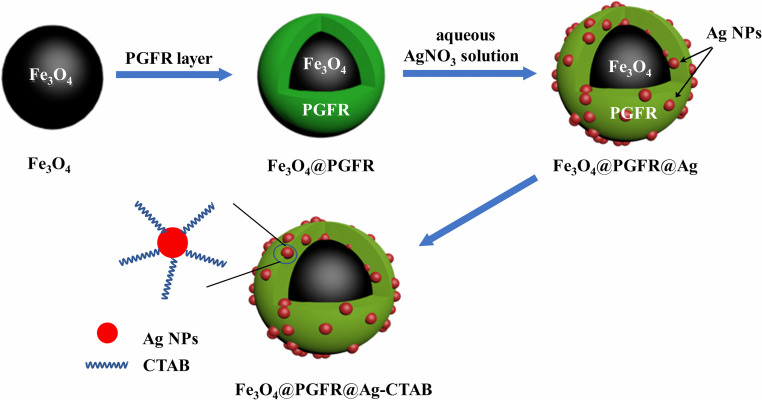
Synthesis route for Fe_3_O_4_@PGFR@Ag-CTAB core–shell nanomaterials.

The preparation procedure of the Fe_3_O_4_@PGFR@Ag nanomaterial is as follows. The prepared Fe_3_O_4_@PGFR (25 mg) was dispersed in 50 mL of an aqueous silver nitrate (2 mM) solution and stirred at room temperature in a dark environment for 2 hours. Fe_3_O_4_@PGFR@Ag was washed several times with deionized water and ethanol, magnetically separated, and dried under vacuum for 6 hours. The charge conversion of Fe_3_O_4_@PGFR@Ag NPs was achieved by immersing Fe_3_O_4_@PGFR@Ag in a CTAB solution for 24 hours (named as Fe_3_O_4_@PGFR@Ag-CTAB). The synthesis route is shown in Fig. 1.

### 2.5 Characterization

The transmission electron microscopy (TEM) images were obtained using JEM-2100F. Scanning electron microscopy (SEM) images were obtained using a Zeiss Merlin compact field-emission instrument at an accelerating voltage of 20 kV. The Fourier-transform infrared (FTIR) spectra were recorded on a Bruker Equinox 55 spectrometer in the transmission mode in the scan range from 4000 to 500 cm^−1^. The UV-vis absorption spectra were recorded on a UV-3600 spectrophotometer (Shimadzu, Japan). The zeta potentials were measured using a NanoBrook 90Plus Zeta nanograin-sized analyzer (Brookhaven, USA). The magnetic characteristics of the samples were studied using a vibrating sample magnetometer (VSM) (HH-20, China) at an applied field between −1500 and 1500 Oe at room temperature. The X-ray diffraction (XRD) patterns were measured using an XRD-6000 X-ray diffractometer (Shimadzu, Japan). X-ray photoelectron spectroscopy (XPS) results were recorded on an Axis Ultra DLD system using Al Kα radiation.

### 2.6 Catalytic performance test

Initially, an aqueous solution of Fe_3_O_4_@PGFR@Ag was prepared at a concentration of 0.25 mg mL^−1^. Subsequently, the aqueous Fe_3_O_4_@PGFR@Ag solution (200 μL) was added to a mixed solution containing 2 mL of RhB (10 mg mL^−1^) and 1 mL of a freshly prepared sodium borohydride (0.5 M) solution. The concentration change of the solution was monitored using a UV-vis spectrometer. The catalytic activity of Fe_3_O_4_@PGFR@Ag for RhB was evaluated using a quasi-level kinetic equation. The same procedure was followed for methyl orange and tetracycline. To assess the catalytic cyclability of Fe_3_O_4_@PGFR@Ag, an aqueous solution of the contaminant (1 mg mL^−1^, 20 μL) was added to the reaction system for the next catalytic cycle. The catalytic performance of Fe_3_O_4_@PGFR@Ag-CTAB NPs was evaluated using the same method.

## 3. Results and discussion

### 3.1 Characterization of Fe_3_O_4_@PGFR, Fe_3_O_4_@PGFR@Ag, and Fe_3_O_4_@PGFR@Ag-CTAB

**Fig. 2:**
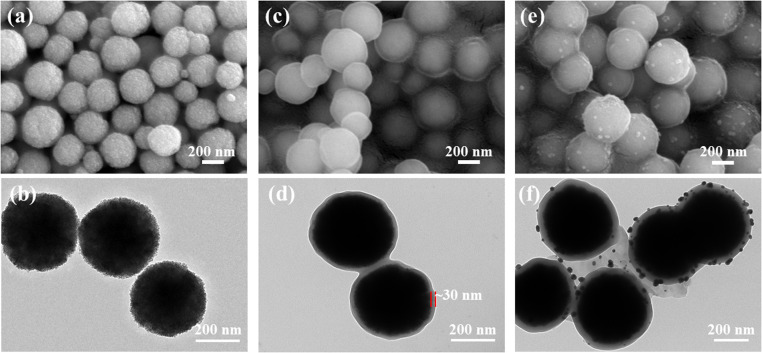
SEM and TEM images. (a) SEM image of Fe_3_O_4_, (b) TEM image of Fe_3_O_4_, (c) SEM image of Fe_3_O_4_@PGFR, (d) TEM image of Fe_3_O_4_@PGFR, (e) SEM image of Fe_3_O_4_@PGFR@Ag, and (f) TEM image of Fe_3_O_4_@PGFR@Ag.

The SEM and TEM images of Fe_3_O_4_, Fe_3_O_4_@PGFR, and Fe_3_O_4_@PGFR@Ag are presented in Fig. 2. As shown in Fig. 2a, Fe_3_O_4_ NPs exhibited a regular and uniform spherical structure with a relatively rough surface. After coating Fe_3_O_4_ NPs with PGFR (Fig. 2c), Fe_3_O_4_@PGFR core–shell nanoparticles were formed, with PGFR uniformly coating the surface of Fe_3_O_4_ NPs. The surface of Fe_3_O_4_@PGFR became smooth, and the thickness of the PGFR layer was approximately 30 nm (Fig. 2d). The TEM image of Fe_3_O_4_@PGFR@Ag (Fig. 2f) revealed that Ag NPs were successfully captured and immobilized within the shell of PGFR. This suggests that the phenolic hydroxyl groups in the PGFR layer can form coordination complexes with silver ions and reduce them to silver nanoparticles, which then grow on the PGFR shell.

**Fig. 3:**
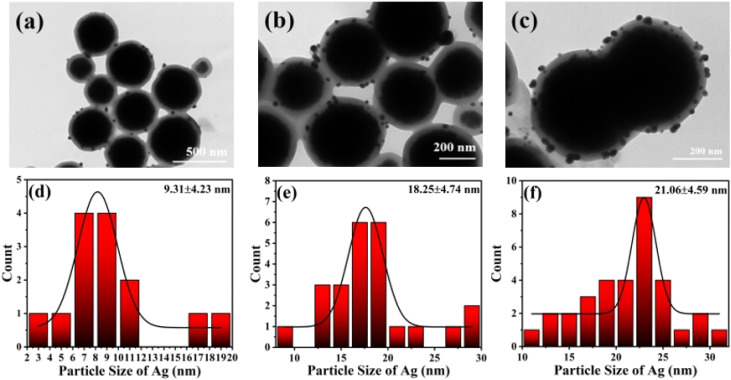
TEM images of (a) Fe_3_O_4_@PGFR@Ag-0.5 mM, (b) Fe_3_O_4_@PGFR@Ag-1 mM, and (c) Fe_3_O_4_@PGFR@Ag-2 mM. (d) Particle size distribution of Ag NPs-0.5 mM, (e) particle size distribution of Ag NPs-1 mM, and (f) particle size distribution of Ag NPs-2 mM.

**Fig. 4:**
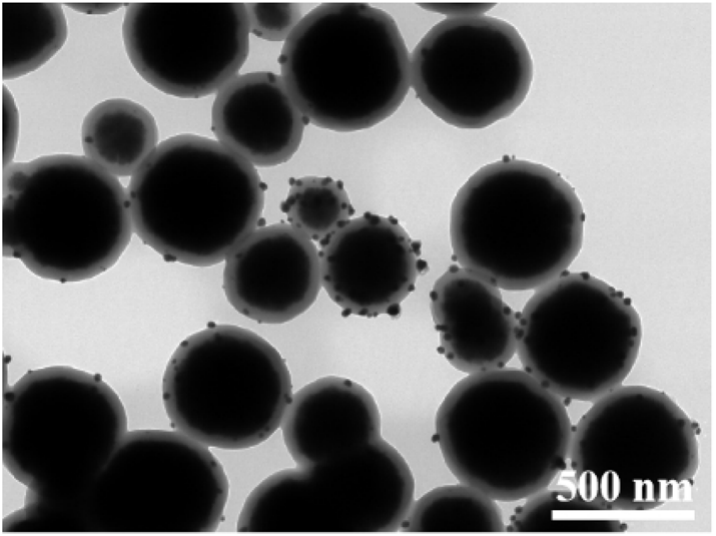
Overall size distribution of Fe_3_O_4_@PGFR@Ag-CTAB.

The TEM images of Fe_3_O_4_@PGFR@Ag prepared with different silver nitrate contents and the Ag^0^ particle size distributions are shown in Fig. 3. The results indicate that the particle size of Ag NPs increased with an increase in the AgNO_3_ dosage. With an increase in the dosage of AgNO_3_, a higher amount of Ag^+^ diffused into the shell layer of PGFR, where it was captured and anchored for reduction deposition. It is also owing to its adhesion properties that Fe_3_O_4_@PGFR@Ag-CTAB aggregation occurs. The overall size distribution of Fe_3_O_4_@PGFR@Ag-CTAB is shown in Fig. 4.

**Fig. 5:**
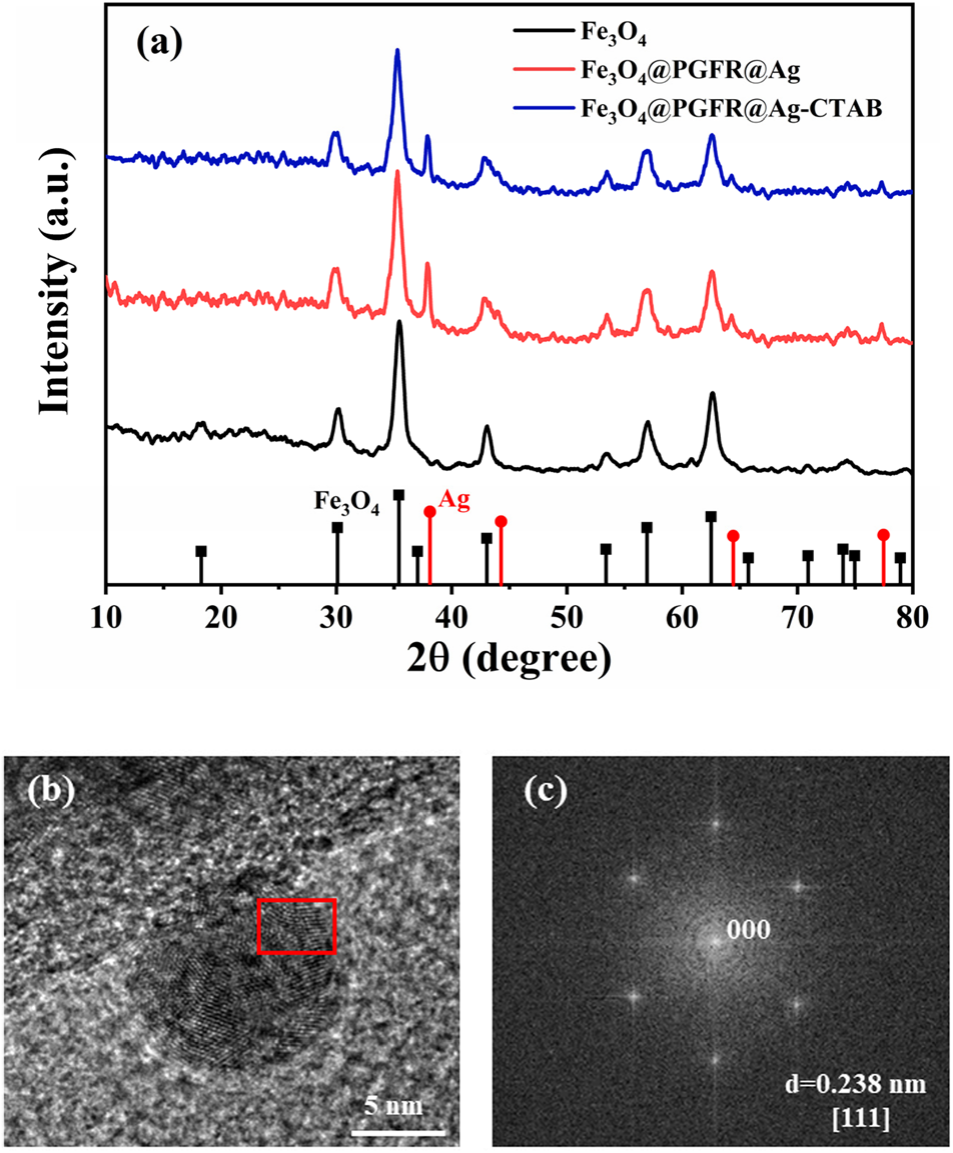
(a) XRD patterns of Fe_3_O_4_, Fe_3_O_4_@PGFR@Ag, and Fe_3_O_4_@PGFR@Ag-CTAB. (b) HRTEM image of Fe_3_O_4_@PGFR@Ag. (c) FFT of Fe_3_O_4_@PGFR@Ag.

The X-ray diffraction (XRD) patterns of Fe_3_O_4_, Fe_3_O_4_@PGFR@Ag, and Fe_3_O_4_@PGFR@Ag-CTAB nanomaterials are shown in Fig. 5. The seven peaks at 18.3°, 30.1°, 35.8°, 43.1°, 54.4°, 57.0°, and 62.6° corresponded to the (1 1 1), (2 2 0), (3 1 1), (4 4 0), (4 2 2), (5 1 1), and (4 4 0) planes of Fe_3_O_4_, respectively (JCPDS card no. 19-0629). The XRD pattern of the Fe_3_O_4_@PGFR@Ag nanohybrid material also confirmed the presence of Ag NPs with four new peaks at 38.1°, 44.3°, 64.4°, and 77.5°, corresponding to the (111), (200), (220), and (311) planes of Ag NPs, respectively (JCPDS card no. 04-0783). The XRD patterns of Fe_3_O_4_@PGFR@Ag and Fe_3_O_4_@PGFR@Ag-CTAB did not differ significantly. This indicates that surface modification by CTAB does not affect the crystalline shape and the presence of Ag NPs. Ag NPs can still maintain their good catalytic activity. The HRTEM images of Ag NPs are shown in Fig. 5b. According to Image J calculations, the lattice spacing of Ag NPs was 0.238 nm (Fig. 5c), corresponding to the (111) crystal plane of the face-centered cubic (fcc) structure. The diffraction spots (Fig. 5c) are the FFT of the areas marked by the red box in Fig. 5b.

**Fig. 6:**
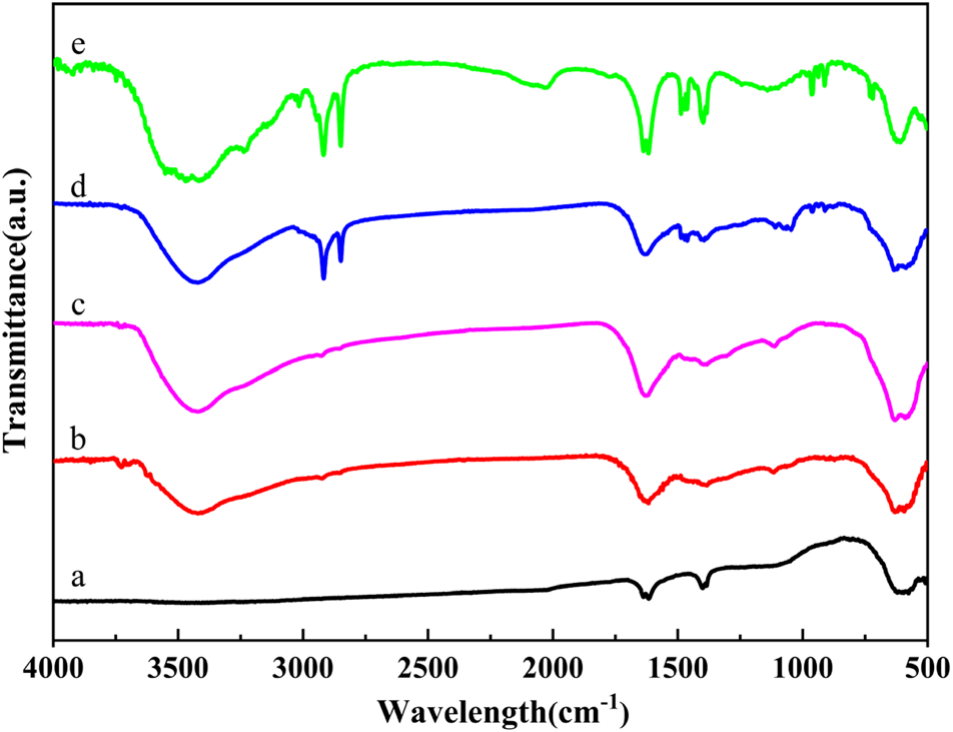
FT-IR spectra of (a) Fe_3_O_4_, (b) Fe_3_O_4_@PGFR, (c) Fe_3_O_4_@PGFR@Ag, (d) Fe_3_O_4_@PGFR@Ag-CTAB, and (e) CTAB.

FT-IR spectroscopy was employed to investigate the chemical structure of Fe_3_O_4_@PGFR@Ag-CTAB core–shell materials. The spectra of the samples are presented in Fig. 6. The peak observed at 580 cm^−1^ corresponded to the stretching vibration of the Fe–O bond in pure Fe_3_O_4_ NPs (Fig. 6a). The two peaks observed at 1305 cm^−1^ and 1112 cm^−1^ corresponded to the stretching vibrations of the C–O bond and the C–O–C vibrational mode, respectively, indicating the presence of the PGFR coating on Fe_3_O_4_ NPs. As shown in Fig. 6c and d, Fe_3_O_4_@PGFR@Ag-CTAB exhibited four additional distinct peaks at 910 cm^−1^, 957 cm^−1^, 2845 cm^−1^, and 2913 cm^−1^ compared to Fe_3_O_4_@PGFR@Ag. The peaks at 910 cm^−1^ and 957 cm^−1^ were attributed to the in-plane C–H bending vibrations, while the peak at 2845 cm^−1^ corresponded to the stretching vibration of –CH_2_. The peak at 2913 cm^−1^ was assigned to the C–H stretching vibration of the saturated carbon of the CTAB end group (–CH_3_). These results confirm the successful preparation of the Fe_3_O_4_@PGFR@Ag-CTAB nanohybrid materials.

**Fig. 7:**
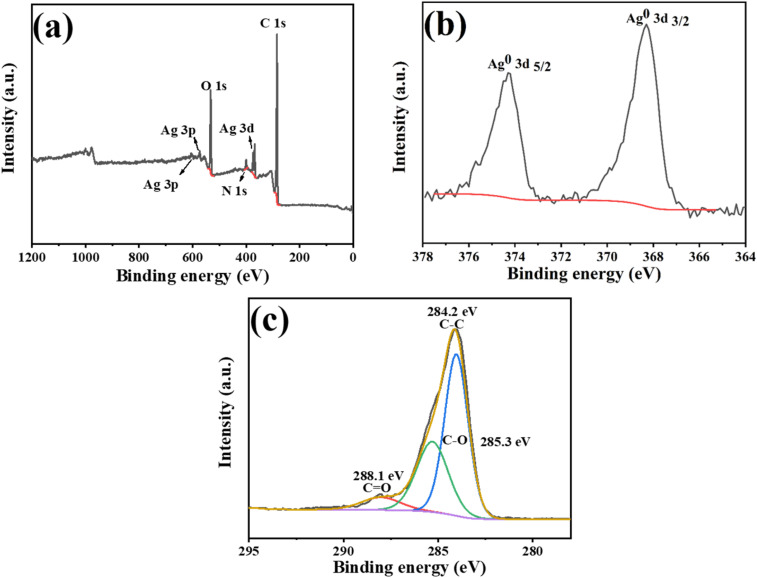
XPS spectra of Fe_3_O_4_@PGFR@Ag: (a) full-scan, (b) Ag 3d, and (c) C 1s spectra.

To further demonstrate the presence of silver as a monomer in the hybrid material, the X-ray photoelectron spectroscopy (XPS) spectra of Fe_3_O_4_@PGFR@Ag nanomaterials were recorded and are presented in Fig. 7. The full-scan XPS spectra of the samples showed the presence of the O 1s orbital, C 1s orbital, and Ag 3d orbital. The high-resolution XPS spectrum of Ag 3d in Fig. 7b revealed that the 3/2 and 5/2 peaks of the Ag 3d orbital were located at 374.2 eV and 368.3 eV, respectively. Previous reports have shown that the single silver peak corresponds to a binding energy of around 370 eV, which is typical for metallic silver. This confirms that the metallic silver presented in the Fe_3_O_4_@PGFR@Ag nanohybrid material is the active material required for subsequent catalysis. XPS further confirmed that silver in the hybrid material existed as a monomer. Fig. 7c shows three peaks based on the C 1s fitted deconvolution, with C 1s binding energies at 284.2 eV, 285.5 eV, and 288.0 eV, corresponding to the C–C bond, C–O bond, and C=O double bond of PGFR, respectively. The TEM image (Fig. 2f) demonstrated the successful encapsulation of Fe_3_O_4_ by the PGFR layer. Ag NPs were reduced by capturing Ag^+^ using the phenolic hydroxyl group, which facilitated the growth of Ag NPs on the PGFR shell layer.

**Fig. 8:**
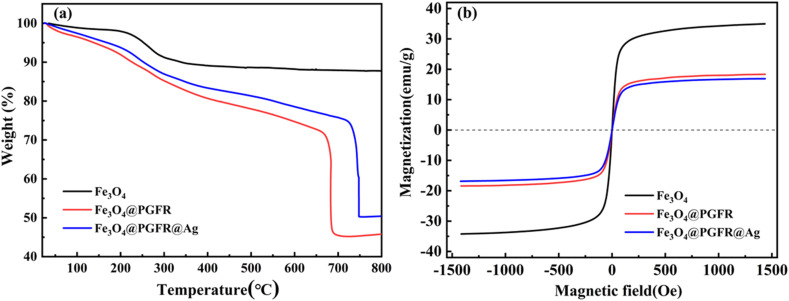
(a) TG curves of Fe_3_O_4_, Fe_3_O_4_@PGFR, and Fe_3_O_4_@PGFR@Ag. (b) VSM curves of Fe_3_O_4_, Fe_3_O_4_@PGFR and Fe_3_O_4_@PGFR@Ag.

The thermogravimetric (TG) curves of Fe_3_O_4_, Fe_3_O_4_@PGFR, and Fe_3_O_4_@PGFR@Ag nanomaterials are presented in Fig. 8a. Catalytic reactions often occur in complex environments, such as high temperatures and heat, and the thermal stability of materials can have a significant impact on their catalytic performance. As shown in Fig. 8a, when the temperature was below 160 °C, the weight loss of Fe_3_O_4_ nanoparticles was due to the evaporation of residual water and organic solvents in the material during the heating process. In the temperature range from 200 °C to 270 °C, the weight loss of the sample was attributed to thermal decomposition and carbonization caused by the thermal degradation and carbonization of trisodium citric acid, which served as a stabilizer for iron tetroxide. For Fe_3_O_4_@PGFR, the approximately 43.5 wt% mass loss was mainly due to the carbonization of the PGFR shell when the temperature increased to around 700 °C. The rapid weight loss of Fe_3_O_4_@PGFR at approximately 700 °C was attributed to the collapse of its core–shell structure.

The mass loss of Fe_3_O_4_@PGFR@Ag was approximately 39.7 wt%, which is lower than that of Fe_3_O_4_@PGFR nanoparticles. As shown in Fig. 8a, Fe_3_O_4_@PGFR and Fe_3_O_4_@PGFR@Ag exhibited similar weight loss stages from 200 °C to 800 °C, but they differed significantly from pure Fe_3_O_4_. This indicates that the PGFR shell layer is successfully encapsulated around Fe_3_O_4_ and Ag NPs are grown *in situ* on the PGFR shell layer. The presence of the PGFR layer had a significant effect on the thermal stability of the catalytic materials. The magnetic properties of the samples were characterized by VSM, as shown in Fig. 8b. The saturation magnetization of pure Fe_3_O_4_ was measured to be 34.5 emu g^−1^. After coating with PGFR, Fe_3_O_4_@PGFR exhibited a reduced magnetization of 18.3 emu g^−1^. Fe_3_O_4_@PGFR@Ag displayed a slight decrease in magnetization (16.9 emu g^−1^). This reduction did not significantly impact the magnetic recovery performance of Fe_3_O_4_@PGFR@Ag, as evidenced by its efficient separation from the solution using an external magnetic field.

**Fig. 9:**
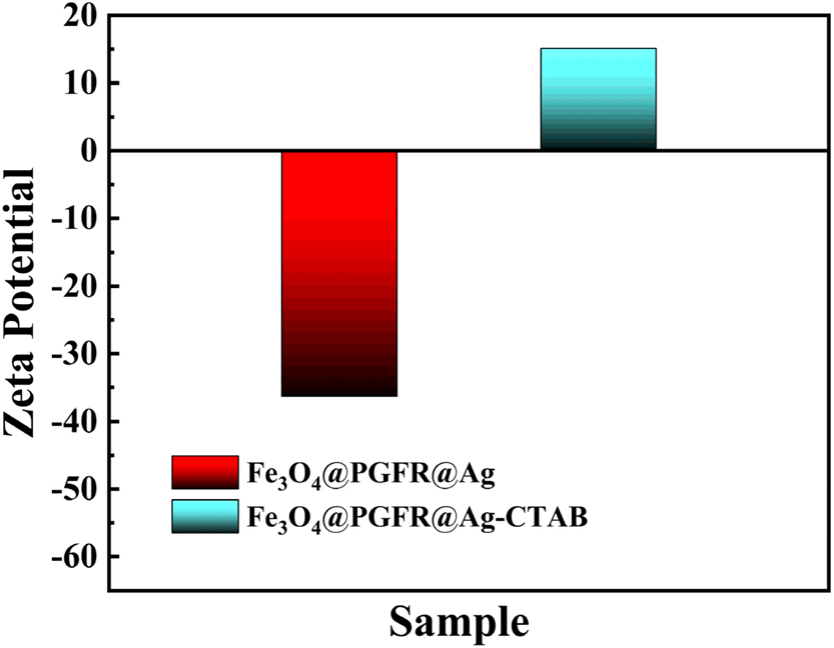
Zeta potentials of Fe_3_O_4_@PGFR@Ag and Fe_3_O_4_@PGFR@Ag-CTAB.

Fig. 9 shows the zeta potentials of Fe_3_O_4_@PGFR@Ag and Fe_3_O_4_@PGFR@Ag-CTAB. The zeta potential of Fe_3_O_4_@PGFR@Ag was approximately −36.3 mV (Fig. 9). Due to the surface electrostatic attraction, a negative surface charge state would exhibit higher catalytic properties for cationic dyes such as RhB. Therefore, changing the surface charge state of the nanomaterial is crucial for preparing nanomaterials with high catalytic performance for anionic dyes such as MO. CTAB, as a cationic surfactant, can combine with metal particles to form noble metal particle-CTAB. This allows the surface charge state of noble metal particles to transition from negative to positive. Fe_3_O_4_@PGFR@Ag-CTAB nanoparticles with a positive surface charge were obtained by modifying Fe_3_O_4_@PGFR@Ag with CTAB. The zeta potential of Fe_3_O_4_@PGFR@Ag-CTAB was approximately 15.1 mV (Fig. 9). The XRD pattern of Fe_3_O_4_@PGFR@Ag-CTAB did not differ significantly from that of Fe_3_O_4_@PGFR@Ag (Fig. 5), indicating that modification with CTAB only changed the surface charge state of the materials without altering the structure of Fe_3_O_4_@PGFR@Ag-CTAB or the presence state of Ag NPs.

### 3.2 Catalytic properties of Fe_3_O_4_@PGFR@Ag and Fe_3_O_4_@PGFR@Ag-CTAB

**Fig. 10:**
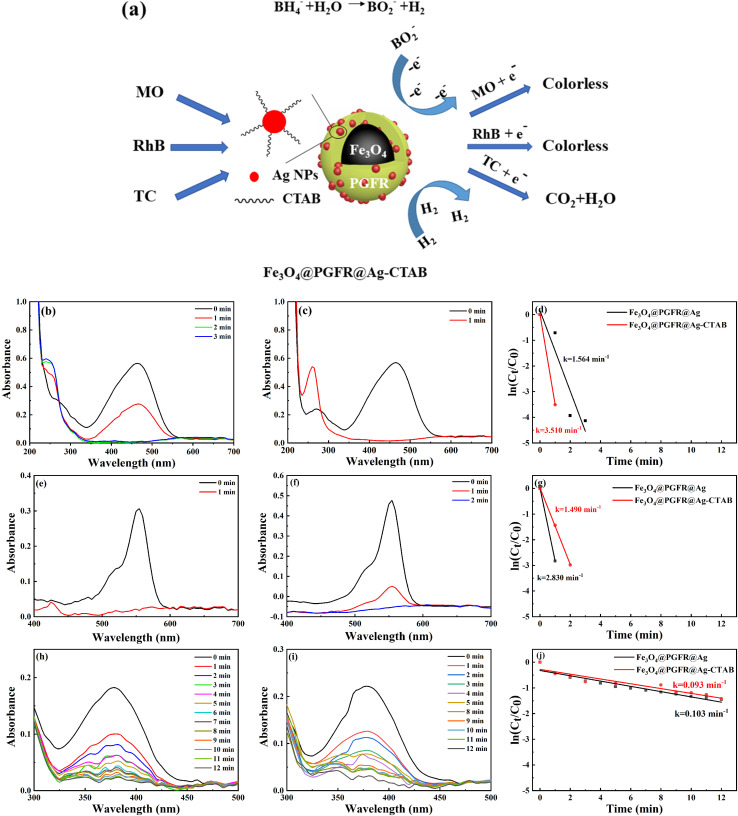
(a) Schematic of the catalytic process. UV-vis spectra for MO: (b) Fe_3_O_4_@PGFR@Ag and (c) Fe_3_O_4_@PGFR@Ag-CTAB. (d) Comparison of first-order rates of reductive degradation. UV-vis spectra for RhB: (e) Fe_3_O_4_@PGFR@Ag and (f) Fe_3_O_4_@PGFR@Ag-CTAB. (g) Comparison of first-order rates of reductive degradation. UV-vis spectra for TC: (h) Fe_3_O_4_@PGFR@Ag and (i) Fe_3_O_4_@PGFR@Ag-CTAB. (j) Comparison of first-order rates of reductive degradation.

Silver nanoparticles have been reported to exhibit excellent catalytic activity and good selectivity towards organic dyes. Therefore, we investigated the catalytic performance of Fe_3_O_4_@PGFR@Ag and Fe_3_O_4_@PGFR@Ag-CTAB towards MO, RhB, and TC in the presence of NaBH_4_. Fig. 10a shows a schematic diagram of the catalytic process for the three substrate models. In brief, the catalysis of the three substrates, which are enriched on the surface of Ag NPs, is achieved *via* the transfer of free electrons of Ag NPs. The UV-vis spectra of MO catalyzed by Fe_3_O_4_@PGFR@Ag are presented in Fig. 10b. It can be observed that after the addition of 200 μL of the Fe_3_O_4_@PGFR@Ag catalyst into the catalytic system, the intensity of the characteristic peak of MO at 465 nm began to decrease, and the intensity of the characteristic peak decreased with increasing catalytic time. When the catalyst was added for approximately 3 minutes, the peak of MO at 465 nm disappeared completely, indicating that the catalytic reaction was completed and MO had been reduced. The catalytic rate of Fe_3_O_4_@PGFR@Ag for MO was 1.564 min^−1^. When 200 μL of the Fe_3_O_4_@PGFR@Ag-CTAB catalyst was added to the MO solution, the characteristic peak of MO at 465 nm rapidly decreased and disappeared within approximately 1 minute. The catalytic rate of Fe_3_O_4_@PGFR@Ag-CTAB for MO was 3.510 min^−1^. These results demonstrate that positively charged Fe_3_O_4_@PGFR@Ag-CTAB significantly enhances the catalytic rate for the anionic dye MO. The MO molecule is enriched on the surface of Ag NPs due to electrostatic effects. This facilitates the rapid acceptance of free electrons generated from the hydrolysis of NaBH_4_, which is attacked by free electrons, and the N=N bonds break to form N–H bonds.

To further analyze the effect of the surface charge state of the catalytic material on its catalytic performance, we conducted comparative catalytic experiments using the cationic dye RhB. As shown in Fig. 10e, the intensity of the characteristic peak of RhB at 550 nm decreased rapidly after the catalyst was added. Due to the electrostatic effect, the cationic dye RhB was more easily enriched on the surface of Fe_3_O_4_@PGFR@Ag with a negative surface charge. The cationic dye RhB was catalyzed, and new RhB molecules were re-attracted to the surface of Ag NPs, reaching a kinetic equilibrium. The catalytic rate *k* was 2.830 min^−1^, according to the fitting of the first-order kinetic equation. For Fe_3_O_4_@PGFR@Ag-CTAB (Fig. 10f), the positive surface charge was retarded due to the aggregation of RhB molecules on Ag NPs, which delayed the time required for RhB molecules to reach the Ag NP surface. The catalytic rate constant (*k*) was calculated to be 1.490 min^−1^; notably, this value was lower than that of Fe_3_O_4_@PGFR@Ag (*k* = 2.830 min^−1^). The catalytic rate constants for the three pollutants are presented in Table 1. The surface charge state of the samples had a significant impact on the catalytic performance of the pollutants. In the case of catalysts and contaminants with opposite charges, the contaminants tended to accumulate around the catalyst owing to electrostatic attraction and subsequently undergo further decolorization and degradation by Ag. However, when the charge state of the catalyst and the contaminant was the same, the contaminants were less likely to accumulate around the catalyst owing to electrostatic repulsion, and the catalytic reaction rate constant was significantly reduced.

**Table 1:** Catalytic rates of the samples for MO, RhB, and TC

Sample	*k* for RhB (min^−1^)	*k* for MO (min^−1^)	*k* for TC (min^−1^)
Fe_3_O_4_@PGFR@Ag	2.830	1.564	0.103
Fe_3_O_4_@PGFR@Ag-CTAB	1.490	3.510	0.093

To evaluate the catalytic performance of Fe_3_O_4_@PGFR@Ag and Fe_3_O_4_@PGFR@Ag-CTAB, neutral tetracycline (TC) was selected as a test substrate. As shown in Fig. 10h, the intensity of the characteristic peak at 380 nm decreased with the progress of the reaction and disappeared after approximately 12 minutes. Compared with Fe_3_O_4_@PGFR@Ag, the catalytic capacity of Fe_3_O_4_@PGFR@Ag-CTAB for TC did not differ significantly. According to the fitting calculation of the first-order kinetic equation (Fig. 10j), the catalytic rate constants of Fe_3_O_4_@PGFR@Ag nanoparticles and Fe_3_O_4_@PGFR@Ag-CTAB nanoparticles for TC were 0.103 min^−1^ and 0.093 min^−1^, respectively (Table 1). These results indicate that switching the material surface charge has little effect on the catalytic performance of neutral TC.

The catalytic properties of various catalytic materials for MO, RhB, and TC are summarized in Tables 2–4, respectively. Compared to the catalysts reported in previous studies, Fe_3_O_4_@PGFR@Ag and Fe_3_O_4_@PGFR@Ag-CTAB exhibited superior catalytic performance.

**Table 2:** Comparison of the TC catalytic degradation performance of different catalytic materials

Sample	TC	Catalyst dosage	*k* (min^−1^)	Ref.
Fe_3_O_4_/BiVO_4_/CdS	10 mg L^−1^	100 mg	0.023	37
Fe_3_O_4_/CuO/C	50 mg L^−1^	300 mg	0.923	38
Bi_2_O_3_ QDs/g-C_3_N_4_	10 mg L^−1^	50 mg	0.014	39
Ag-g-C_3_N_4_	20 mg L^−1^	50 mg	0.041	40
AgI/Ag/Cu-BTC	5 mg L^−1^	80 mg	0.022	5
Fe_3_O_4_@PGFR@Ag-CTAB	2 mL, 10 mg L^−1^	0.25 mg mL^−1^, 200 μL (0.05 mg)	0.103	This work

**Table 3:** Comparison of the MO catalytic degradation performance of different catalytic materials

Sample	MO	Catalyst dosage	*k* (min^−1^)	Ref.
GO/TiO_2_/Fe_3_O_4_	50 mL, 10 mg L^−1^	60 mg	0.022	41
Au-TA	50 mL, 8 mg L^−1^	50 μL	0.005	42
Ag-TA	50 mL, 8 mg L^−1^	50 μL	0.59	42
Fe_3_O_4_@C@Au-CTAB	0.25 mg L^−1^	1 mg	1.870	25
Fe_3_O_4_@PGFR@Ag-CTAB	2 mL, 10 mg L^−1^	0.25 mg mL^−1^, 200 μL (0.05 mg)	3.510	This work

**Table 4:** Comparison of the RhB catalytic degradation performance of different catalytic materials

Sample	RhB	Catalyst dosage	*k* (min^−1^)	Ref.
F-CeO_2_/CdS	50 mL, 10 mg L^−1^	50 mg	0.036	43
BN/C_3_N_4_	50 mL, 5 mg L^−1^	100 mg	0.072	44
Pd-TiO_2_/Bi_2_O_3_	10 mg L^−1^	50 mg	0.110	45
Fe_3_O_4_@C@Au-CTAB	0.25 mg L^−1^	1 mg	0.500	25
Fe_3_O_4_@PGFR@Ag-CTAB	2 mL, 10 mg L^−1^	0.25 mg mL^−1^, 200 μL (0.05 mg)	0.103	This work

### 3.3 Stability and recyclability

**Fig. 11:**
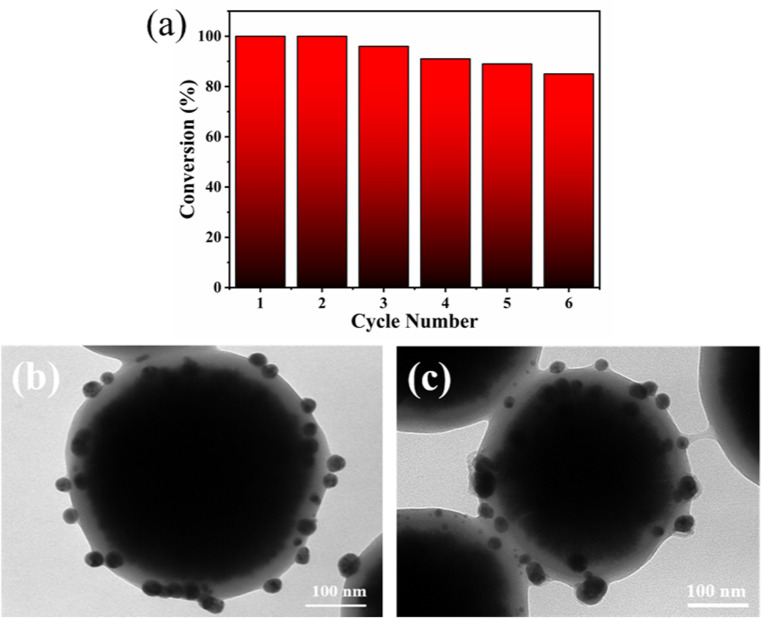
Cycle performance. (a) Catalytic reuse efficiency. TEM images of Fe_3_O_4_@PGFR@Ag-CTAB (b) before the cycle and (c) after six cycles.

To assess the stability and recyclability of the catalytic materials, Fe_3_O_4_@PGFR@Ag-CTAB was employed to catalyze MO. Six cycling experiments were carried out. As shown in Fig. 11a, Fe_3_O_4_@PGFR@Ag-CTAB nanoparticles exhibited an efficient catalytic capacity for MO, which was sustained at approximately 85%. This indicated that Fe_3_O_4_@PGFR@Ag-CTAB nanoparticles possessed an effective cycling capacity and stability for MO. Furthermore, as shown in Fig. 11c, after multiple cycles, the Ag NPs in Fe_3_O_4_@PGFR@Ag-CTAB nanoparticles did not exhibit significant shedding and the structure remained intact. The sustainability of catalytic materials was confirmed by the presence of silver nanoparticles and the integrity of the core–shell structure. The cyclic tests demonstrated that PGFR was an ideal carrier for Ag NPs, which could achieve the efficient and continuous chelation and anchoring of Ag NPs. The CTAB-modified Fe_3_O_4_@PGFR@Ag nanoparticles maintained efficient catalytic activity for MO in cycling tests, and the structure of the catalytic materials remained intact during the reaction process.

## 4. Conclusions

In this work, Fe_3_O_4_@PGFR@Ag core–shell nanomaterials were successfully prepared with controlled charge switching using a facile CTAB modification method. Fe_3_O_4_@PGFR@Ag-CTAB exhibited efficient catalytic properties towards the anionic dye MO, which was attributed to favorable electrostatic interactions. The catalytic rate constant of Fe_3_O_4_@PGFR@Ag-CTAB for MO was found to be 3.510 min^−1^, 124% higher than that of Fe_3_O_4_@PGFR@Ag. Similarly, the *k* value for the cationic dye RhB of Fe_3_O_4_@PGFR@Ag was 2.83 min^−1^, which was 90% higher than that of positively charged Fe_3_O_4_@PGFR@Ag-CTAB. Moreover, Fe_3_O_4_@PGFR@Ag and Fe_3_O_4_@PGFR@Ag-CTAB showed similar catalytic degradation efficiencies towards the neutral antibiotic TC. These results demonstrated that the catalytic activity was not only dependent on the property of the catalyst but also on the charge state of the contaminant.

## Author contributions

Liping Jiang: data curation, formal analysis, methodology, and writing – original draft. Yang Xi: data curation, formal analysis, and writing – review & editing. Ziyi Xu: investigation and methodology. Zewen Song: investigation and methodology. Yuwei Cui: formal analysis and investigation. Haijun Zhou: data curation, funding acquisition, project administration, supervision, and writing – review & editing.

## Conflicts of interest

The authors declare that they have no known competing financial interests or personal relationships that could have appeared to influence the work reported in this paper.

## Data Availability

The authors affirm that the data supporting the findings of this study are included in the article. Additional data can be made available from the corresponding author upon reasonable request.
